# Chronic Restraint Stress Upregulates Erythropoiesis through Glucocorticoid Stimulation

**DOI:** 10.1371/journal.pone.0077935

**Published:** 2013-10-18

**Authors:** Jeffrey L. Voorhees, Nicole D. Powell, Leni Moldovan, Xiaokui Mo, Timothy D. Eubank, Clay B. Marsh

**Affiliations:** 1 The Dorothy M. Davis Heart and Lung Research Institute, Division of Pulmonary, Allergy, Critical Care, and Sleep Medicine, The Ohio State University, Columbus, Ohio, United States of America; 2 Department of Internal Medicine, The Ohio State University, Columbus, Ohio, United States of America; 3 Section of Oral Biology, College of Dentistry, The Ohio State University, Columbus, Ohio, United States of America; 4 Center for Biostatistics, The Ohio State University, Columbus, Ohio, United States of America; University of Rochester, United States of America

## Abstract

In response to elevated glucocorticoid levels, erythroid progenitors rapidly expand to produce large numbers of young erythrocytes. Previous work demonstrates hematopoietic changes in rodents exposed to various physical and psychological stressors, however, the effects of chronic psychological stress on erythropoiesis has not be delineated. We employed laboratory, clinical and genomic analyses of a murine model of chronic restraint stress (RST) to examine the influence of psychological stress on erythropoiesis. Mice exposed to RST demonstrated markers of early erythroid expansion involving the glucocorticoid receptor. In addition, these RST-exposed mice had increased numbers of circulating reticulocytes and increased erythropoiesis in primary and secondary erythroid tissues. Mice also showed increases in erythroid progenitor populations and elevated expression of the erythroid transcription factor KLF1 in these cells. Together this work reports some of the first evidence of psychological stress affecting erythroid homeostasis through glucocorticoid stimulation.

## Introduction

Under homeostatic conditions the body produces erythrocytes at a rate sufficient to compensate for normal red blood cell turnover. However, in response to elevated glucocorticoid levels, erythroid progenitors rapidly expand to produce large numbers of young erythrocytes. This process is subject to the influence of many humoral factors, chief among them are erythropoietin (Epo) and glucocorticoids. Epo and glucocorticoids are both essential for normal erythropoiesis. In normoxia, constitutive expression of Epo facilitates erythropoiesis. However, under hypoxic conditions resulting from limited oxygen availability, blood loss, anemia, or acute chemical exposure, elevated Epo expression maintains erythoid populations by facilitating the rapid proliferation and survival of erythroid progenitor cells [1-4]. Glucocorticoids are necessary for erythropoiesis during fetal development as well as for maintenance of homeostatic red cell expression in adults [3,5-8]. Glucocorticoids enhance the formation of murine erythroid colonies [9] and increase erythroid proliferation under conditions of limited Epo [10,11]. Sustained glucocorticoid exposure stimulates proliferation of erythroid progenitors [12,13] and ligand-bound glucocorticoid receptor (GR) acts cooperatively with the transcription factor KLF1 in bi-potent megakaryocyte-erythroid progenitor (MEP) cells to promote terminal erythroid differentiation [14-17]. Taken together, this suggests that sustained elevations in glucocorticoid levels observed in response to psychological stress may enhance erythroid progenitor proliferation and positively influence erythropoiesis.

Previous work demonstrates hematopoietic changes in rodents exposed to physical and psychosocial stressors [18-24]. However, the majority of reports focus on adrenergic and monoaminergic response and those specifically addressing myelopoiesis, finding diminished CFU-GM populations [19-22,24] without addressing the effects of psychological stress on erythroid development. Here we employed clinical, laboratory and genomic analysis of a murine model of chronic restraint stress (RST) to examine the effects of chronic psychological stress on erythropoiesis.

## Methods

### Mice

Female C57BL/6J mice age 6-8 weeks were purchased from The Jackson Laboratory (Bar Harbor, ME) and housed in an all-female room in groups of five per cage in an AAALAC-accredited facility on a 12-hour (0600/1800h) light/dark cycle with *ad libitum* access to standard rodent chow and water. Female mice were selected due to lower incidences of injurious physical interactions. Mice were allowed to acclimate for 7-10 days before exposure to experimental procedures outlined in a protocol approved by The Ohio State University’s Institutional Animal Care and Use Committee and Office of Responsible Research Practices. Mice were handled minimally and humanely throughout the study and no signs of hypothermia or irregular grooming were noted. Mice were humanely sacrificed by CO_2_ asphyxiation.

### Restraint stress

Following an acclimation period of 7-10 days, each mouse receiving restraint stress was placed in an individual well-ventilated 50mL polystyrene tube at 0900h and returned to its respective cage in a horizontal resting position. At 1500h RST animals were removed from restraint tubes and allowed to freely move until the next restraint exposure. Control animals were denied access to food and water during the RST period (0900-1500h) and were otherwise not disturbed. Following the conclusion of the stress period on Day 28, RST and control animals were permitted access to food and water ad libitum.

### Experiment 1 – Erythropoiesis

To examine the physiological effects of restraint stress on erythropoiesis, individual cages of mice were randomly assigned to control or RST groups. Following the acclimation period, mice designated for RST were subjected to restraint stress for up to 28 days. On the mornings of Day 7, 14, 21, 28, 35, and 42 mice were sacrificed and blood was collected by cardiac puncture for RNA expression analysis as well as quantification of circulating reticulocytes, corticosterone and erythropoietin levels. 

### Experiment 2 – Glucocorticoid receptor antagonism

To consider the role of glucocorticoids, individual cages of animals were randomly assigned to control, RST, RST+RU486 (RU486), or RST+vehicle (vehicle) groups. Following the acclimation period, mice designated for RST were subjected to restraint stress as described. Immediately prior to restraint stress exposure on Days 0-20, each mouse in the RU486 groups received a subcutaneous injection of 0.4mg RU486 (approximately 20mg/kg) in 50µL of 50% ethanol, 50% PBS. Vehicle mice received a corresponding daily injection of 50µL of 50% ethanol, 50% PBS immediately prior to RST on Days 0-20.On the morning of Day 21 mice were sacrificed for blood and tissue collection.

### Blood and tissue collection

Mice were euthanized by CO_2_ asphyxiation and immediately weighed. Upon sacrifice blood was collected by cardiac puncture. Anticoagulated whole blood was collected and immediately delivered in EDTA microtainers (Becton Dickinson, Franklin Lakes, NJ) for reticulocyte quantification using a dual-laser automated hematological cytometer (FORCYTE Autosampler 10 Hemotology Analyzer, Oxford Science, Inc., Oxford, CT; Comparative Pathology and Mouse Phenotyping Shared Resource, College of Veterinary Medicine, The Ohio State University, Columbus, Ohio). Serum was isolated using serum separator tubes (BD, Franklin Lakes, NJ). On Day 7 whole blood was collected into RNAlater® as part of Mouse RiboPure™-Blood RNA Isolation Kit (Invitrogen, Carlsbad, CA) and stored at -80°C until RNA isolation per manufacturer’s instructions. On Day 21 serum was isolated from whole blood and stored at -80°C until analysis. Additionally, spleens, thymuses, and adrenal glands were collected and weighed and bone marrow from femuras well as spleen and liver samples were collected andprepared foranalysis.

### Microarray and functional network analysis

mRNA was extracted from the whole blood of RST (n = 4) and control mice (n = 4) sacrificed on Day 7 using a Mouse RiboPure RNA Isolation Kit (Invitrogen, Carlsbad, CA) according to manufacturer’s instructions. Briefly, blood was centrifuged before removal of RNAlater solution and lysis with a guanidinium-based solution. Total RNA was extracted and purified over solid phase silica columns.RNA yields were determined by Nanodrop (ND-1000 Spectrophotometer, NanoDrop Technologies, Wilmington, DE) and RNA integrity was assessed by capillary electrophoresis using an Agilent Bioanalyzer 2100 (Agilent Technologies, Santa Clara, CA). 30ng total RNA from samples with RNA integrity scores >8.0 were reverse-transcribed and amplified using the Ovation™RNA Amplification System (NuGEN Technologies, Inc., San Carlos, CA), the cDNA was purified using DNA Clean & Concentrator kit (Zymo Research Corp. Orange, CA), then biotin-labeled and fragmented using the FL-Ovation™cDNA Biotin Module V2 (NuGEN Technologies). At all steps the quantity and quality of the preparations was controlled using NanoDrop (NanoDrop Technologies, Wilmington, DE) and Bioanalyzer. Mouse Genome 430 2.0 AffymetrixGeneChips® (Affymetrix, Santa Clara, CA) capable of analyzing over 39,000 transcripts were loaded with the fragmented, labeled cDNA and hybridized overnight (18 hrs) at 45°C, 60 rpm, in the hybridization oven (Affymetrix). Washing and staining of the array was performed on the Fluidics Station 400 (Affymetrix) using the appropriate fluidics scripts (Protocol EukGE-WS2.v5) and the reagents from GeneChip® Hybridization Wash and Stain Kit (Affymetrix). The arrays were scanned by the GeneChip® Scanner 3000 (Affymetrix). Data acquisition and preliminary analysis were done with the GeneChip® Operating Software (GCOS) v1.4.0.036, also from Affymetrix. The .cel files were then imported into Partek Genomic Suite (Partek Inc., St. Louis, MO), using GSRMA, which was used for the remaining analyses. Using the presence call obtained in GCOS, we kept for further analysis in Partek those genes that demonstrated a P (present) or M (marginal) call in at least two experiments of the quadruplicate, in at least one set of experimental conditions. On these genes we then performed (a) Principal Component Analysis and (b) ANOVA, followed by False Discovery Rate correction and of those, the 2642 genes with a significance level p < 0.05 and a fold-change < -1.5 and >1.5 were further analyzed. The list of genes meeting these criteria were uploaded with experimental results and corresponding Genbank accession numbers to Ingenuity Pathways Analysis (Ingenuity® Systems, www.ingenuity.com) for functional network analysis. Predictions of influence on individual biological functions or canonical pathways were generated with significance scores [25].

### Analysis of erythrocyte expression

On day 21 bone marrow, spleen, and liver were collected into cold PBS. Tissues were manually disrupted and filtered through 100 µm nylon mesh screens (Fisher Scientific, Pittsburgh, PA) to produce single-cell suspensions. Samples were labeled with fluorochrome-labeled anti-mouse PE-CD71 and FITC-Ter119 (BioLegend, San Diego, CA) for flow cytometry. Cells expressing CD71 and Ter119 were quantified by flow cytometry using an LSRII flow cytometer (BD Biosciences, Franklin Lakes, NJ; Analytical Cytometry Shared Resource, The Ohio State University Comprehensive Cancer Center, Columbus, Ohio). Representative gating, forward and side scatter plots, quantification and flow parameters used in identifying CD71^+^/Ter119^+^ erythrocytes are shown in a supplementary figure (Figure S1).

### Corticosterone quantification

Corticosterone levels were assessed using a corticosterone double antibody ^125^I RIA kit (MP Biomedicals, Costa Mesa, CA) according to manufacturer’s instructions and samples were assessed using a Packard Cobra II Auto Gamma counter (Perkin-Elmer Wellesley, MA). Samples were prepared and assayed according to manufacturer’s instructions using a Quantikine mouse erythropoietin immunoassay kit (R&D Systems, Minneapolis, MN). 

### Erythroid progenitor cell isolation

Bone marrow was collected on day 21 by flushing femurs with RPMI 1640. Single-cell suspensions were prepared by filtering marrow through 100 µm nylon mesh screens (Fisher Scientific, Pittsburgh, PA). Lineage-depleted cells were isolated using a murine-specific MACS Lineage Cell Depletion Kit according to manufacturer’s instructions (Miltenyi, Cologne, Germany). Fluorochrome-labeled anti-mouse antibodies were purchased from BioLegend (San Diego, CA) and lin(-), CD34-, CD16/32low, Sca-1-, c-kit+ megakaryocyte-erythroid progenitor cells [26,27] were isolated by flow cytometry using an Aria III cell sorter (BD Biosceinces, Franklin Lakes, NJ; Analytical Cytometry Shared Resource, The Ohio State University Comprehensive Cancer Center, Columbus, Ohio). Isolated MEP cells were quantified and collected in TriReagent (Sigma, St. Louis, MO) and immediately frozen at -80°.

### Transcription factor analysis

Erythroid progenitor cells isolated from bone marrow were lysed upon collection in TriReagent. Nucleic acid was extracted with chloroform before mRNA purification using an RNeasy Mini Kit (Qiagen, Valencia, CA). Reverse transcription was performed using SuperScript™ II Reverse Transcriptase (Invitrogen, Carlsbad, CA) and comparative quantitation was performed by real-time RT-PCR for each gene using SYBR Green Mastermix with Rox as a reference dye.Standard curves were performed for each transcript and used to determine relative Ct values. cDNA levels were normalized against GAPDH expression in each sample.RT-PCR was performed using ABI 7900 HT system (Applied Biosystems, Carlsbad, CA). Primers are provided in a supplementary table (Figure S2).

### Statistics

Analysis of variance (ANOVA) models were used to analyze the effect of stress, length of stress, and their interaction on the outcomes and assessments were performed separately for stress and post-stress periods. Holm’s method was applied to adjust for multiplicity of the primary outcomes and control the overall family-wise error rate at α = 0.05 [26]. PCR data were subjected to Shapiro-Wilk test using Statistical Analysis Systems (SAS Institute, Inc., Cary, NC) statistical software. Observations greater than interquartile ranges from the first and third quartile were considered outliers and were excluded in the subsequent analysis [27].

## Results

### Functional network analysis predicts disruption of normal hematopoiesis in response to RST

**Figure 1:**
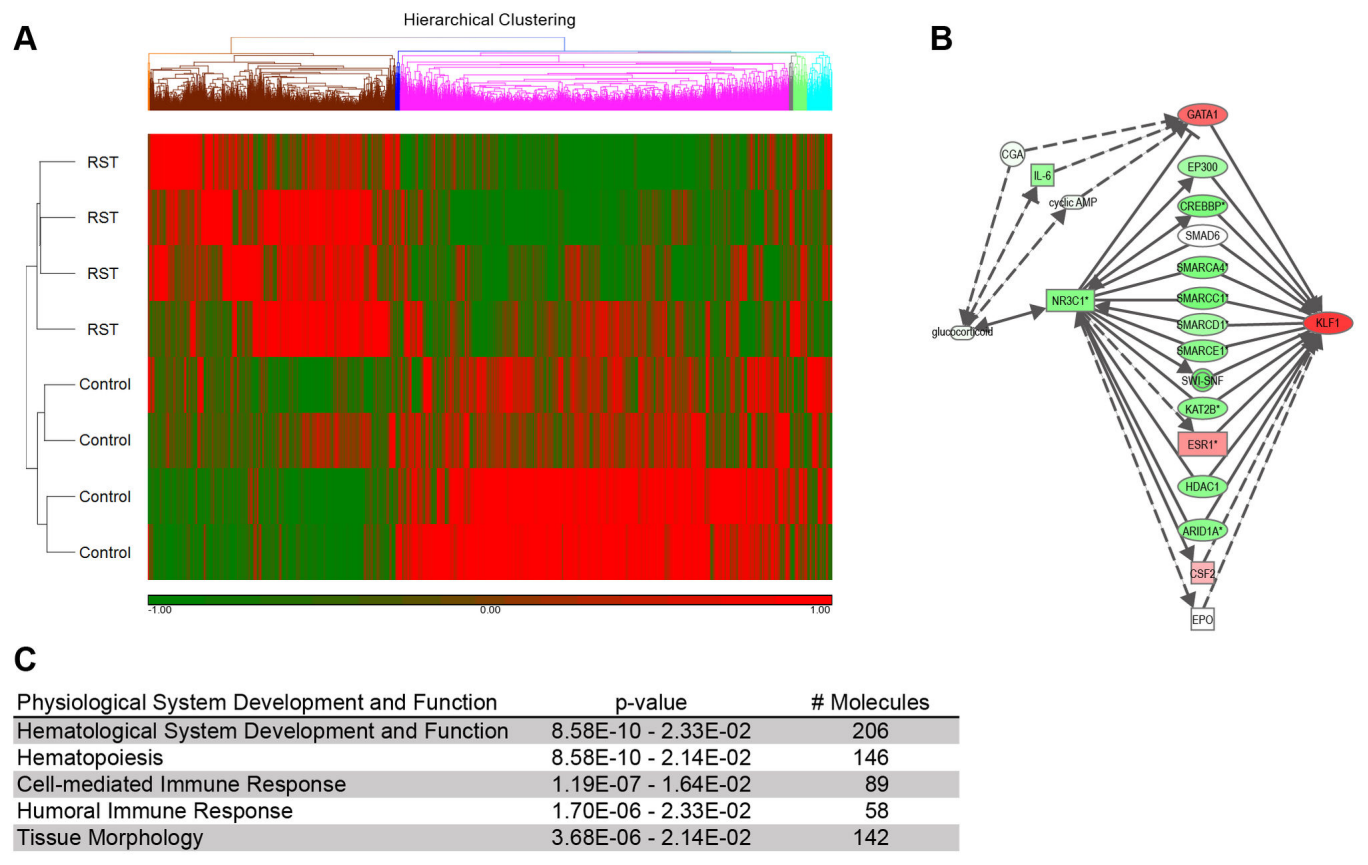
Microarray analysis predicts disrupted hematopoiesis in response to RST. Following seven days of RST, mice were sacrificed and total mRNA was extracted from the whole blood using and purified over solid phase silica columns. mRNA with integrity scores >8.0 was reverse-transcribed and run on Mouse Genome 430 2.0 Affymetrix GeneChips® (control, n = 4 and RST, n = 4). Following internal corrections, genes with significance levels of p < 0.05 and greater than 1.5-fold change were uploaded with corresponding Genbank accession numbers to Ingenuity Pathways Analysis functional network analysis. (**A**) Hierarchical clustering was performed on genes demonstrating statistically significant changes, using Partek Genomic Suite, the Euclidean algorithm with average linkage, and standardized gene expression. (**B**) Ingenuity Pathways Analysis suggest significant disruption of hematopoiesis and hematological function in response to RST. (**C**) Pathway mapping indicates elevated expression of the pro-erythroid transcription factor KLF1 in response to RST and that this change could be affected through the glucocorticoid receptor (NR3C1) In (**A**) and (**C**), green represents down-regulation and red up-regulation of respective gene expression.

 Microarray analyses were conducted as part of characterization of the biological responses to restraint stress. RNA expression profiles of RST and control mice were compared and microarray analyses were conducted using over 45,000 probe sets representing over 34,000 genes and microarray data were uploaded to Gene Expression Omnibus (GSE47725). Hierarchical clustering of genes that displayed significant changes in expression shows clear differences between RST and control mice (Figure **1A**). Predictions of influence on individual biological functions or canonical pathways were generated with significance scores. Functional network analyses suggest significant disruption of hematopoiesis and hematological function in response to RST (Figure **1B**). The microarray revealed a 2.53-fold upregulation in the pre-erythroid transcription factor KLF1 in response to RST and pathway mapping indicated that that this change in expression could be affected through the glucocorticoid receptor (NR3C1) (Figure **1C**).

### RST increases circulating reticulocytes

**Figure 2:**
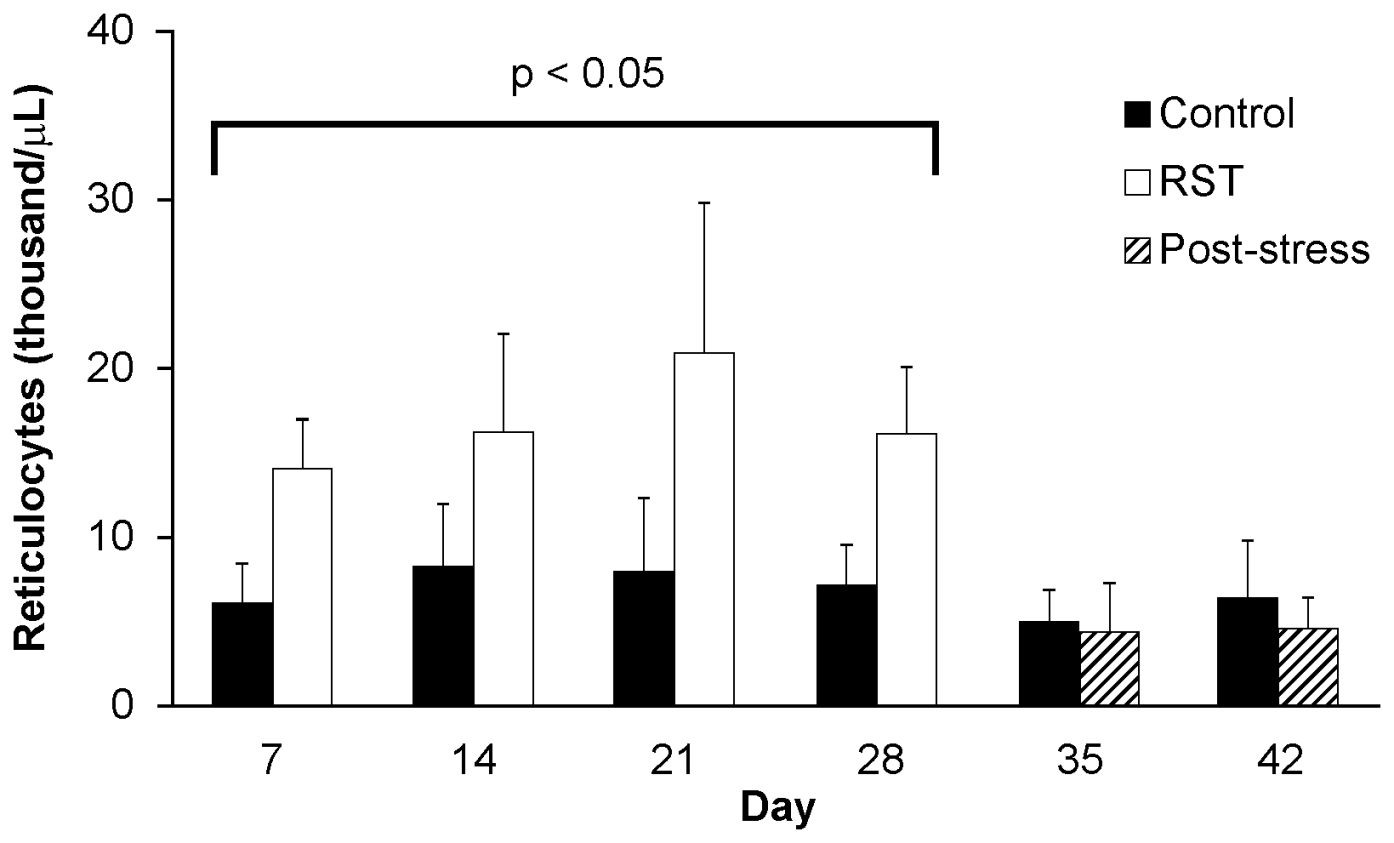
RST increases circulating reticulocytes. A cohort of wildtype female C57Bl/6 mice were left unstressed (Control) or subjected to 6 hrs of restraint stress (RST) for 28 days followed by humane sacrifice and tissue collection. Another cohort of mice was allowed to recover for an additional 14 days without RST (Post-stress) prior to sacrifice and tissue collection. Whole blood was collected via retro-orbital bleeds and circulating reticulocytes quantified during (days 0-28) and following the stress period (days 29-42) using a clinical flow hemocytometer. Data were collected without repeated sampling of individuals. Mice subjected to RST presented significantly higher numbers of reticulocytes compared to unstressed control mice. For each data point n = 9-15 individuals. Data shown is mean +SEM. *, p < .05 by ANOVA vs. unstressed control mice.

To examine the effects of restraint stress on erythropoiesis, circulating levels of reticulocytes were examined during periods of chronic restraint stress as well as in the weeks following stress cessation. Circulating reticulocyte levels were elevated during the restraint period (Day 1-28) and returned to control levels in the post-stress period (**Day 29-42;** Figure **2**).

### RST increases erythropoiesis in primary and secondary hematopoietic organs

**Figure 3:**
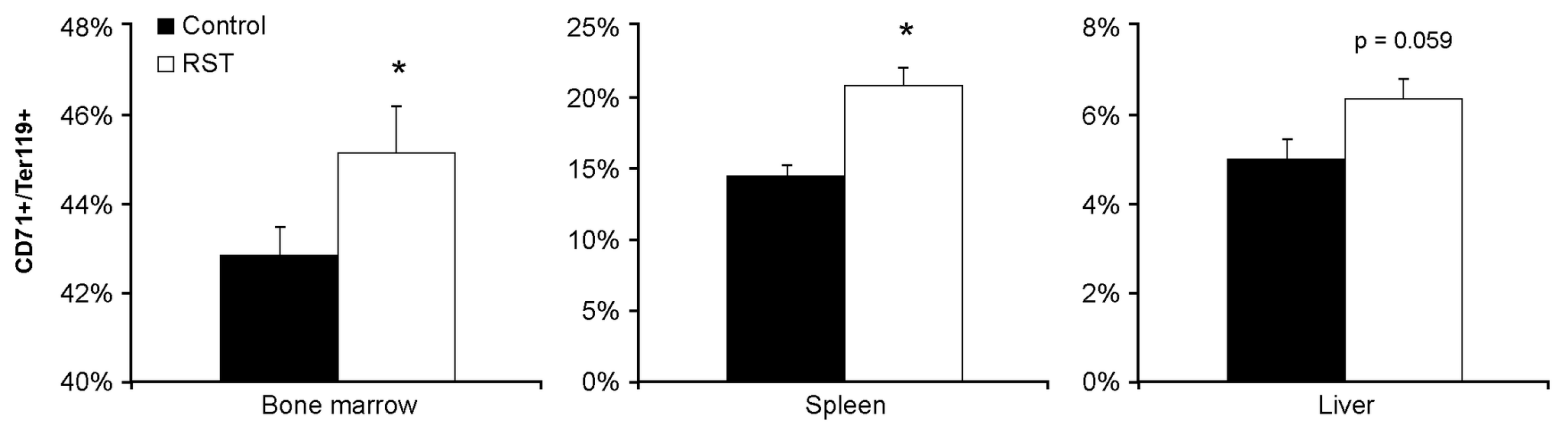
RST elevates erythropoiesis in primary and secondary tissues. From the same experiment in Figure 2, tissue was collected from control mice or mice exposed to RST after 21 days. Cells from bone marrow and homogenized spleens and livers were immunostained with fluorochrome-conjugated antibodies for erythrocyte differentiation (CD71 and Ter119) and quantified by flow cytometry as a percentage of lineage negative cells in primary (Bone marrow) and secondary (Spleen) and (Liver) hematopoietic tissues. RST increased CD71+/Ter119+ cell populations from these tissues compared to tissue from unstressed mice. Data were collected without repeated sampling of individuals. For each data point, n = 5. Data shown is mean +SEM. *, p < 0.05 by Student T-test vs. unstressed control mice in same tissue.

In considering the source of elevated erythrocyte expression, we examined the presence of early terminal differentiated erythrocytes in primary and extramedullary hematopoietic tissues [28]. Elevated numbers of CD71+/Ter119+ erythroblasts were observed in bone marrow, spleen, and liver by day 21 (Figure **3**). These data show increased immature erythrocytes [29-31] and were consistent with observed increases in circulating young erythrocytes.

### RST elevates stress responses

**Figure 4:**
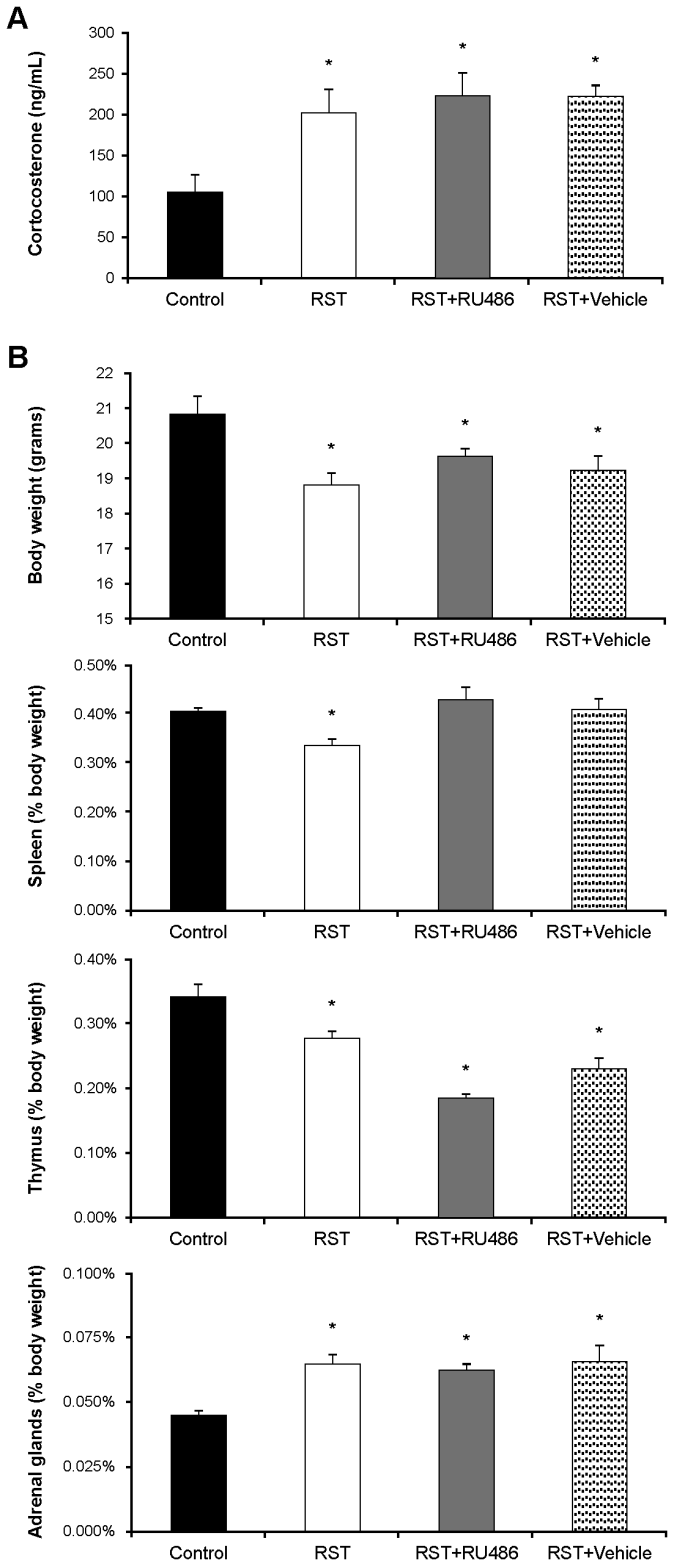
The glucocorticoid receptor antagonist RU486 does not prevent biological stress response. A) Wildtype female C57Bl/6 mice were left unstressed (Control), subjected to 6 hrs of restrain stress (RST), subjected to RST and treated daily with 0.4 mg/day RU486 in 50 µL of 50% ethanol/50% PBS (RST + RU486), or subjected to RST and treated daily with 50 µL of 50% ethanol/50% PBS (RST + Vehicle). RU486 and vehicle treatments were given by subcutaneous injection at 0900h immediately before RST exposure. After 21 days, the mice were sacrificed and whole blood collected via atrial puncture. Serum corticosterone levels were determined using a ^125^I double antibody radioimmunoassay. Serum corticosterone was significantly elevated in all conditions compared to unstressed control mice. For each data point n = 5-8 individuals. Data shown is mean +SEM. *, p < .05 by ANOVA. B) After whole blood collection, total mouse body weight was recorded. Spleen, thymus, and adrenal glands were subsequently collected, weighed, and standardized as a percent of total body weight. Mice subjected to RST, with or without RU486 or vehicle treatment, weighed significantly less than unstressed control mice. The spleens and thymuses from the mice subjected to RST, with or without RU486 or vehicle treatment, were significantly lighter than control mice. In contrast, adrenal glands from mice subjected to RST were significantly heavier than control mice and RU486 or vehicle treatment had no effect. For each data point n = 5-8 individuals. Data shown is mean +SEM. *, p < .05 by ANOVA.

Elevated and sustained stress responses have been described over periods of 28 days using a similar restraint stress protocol [32]. As measures of sustained elevations in stress response we examined circulating corticosterone levels, bodyweight, and spleen, thymus, and adrenal mass. At day 21, mice exposed to RST had elevated baseline corticosterone levels, diminished body weight, diminished spleen mass, decreased thymic mass, and increased adrenal mass (Figure **4**). RU486- and vehicle-treated RST mice had similar sustained elevations in stress markers, though did not have reduced spleen mass. 

### Glucocorticoid receptor antagonism blocks RST-induced elevations in reticulocytes

**Figure 5:**
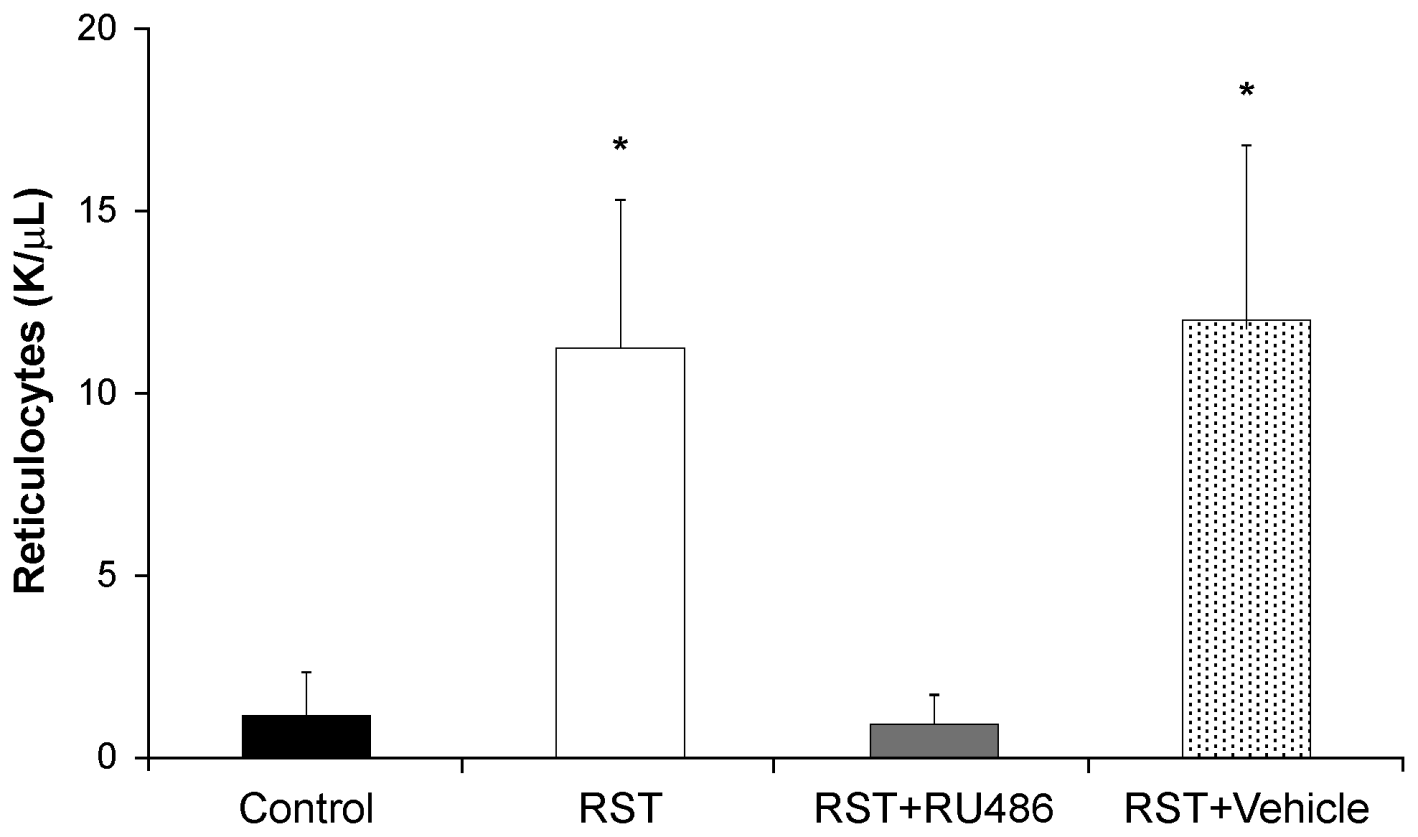
Glucocorticoid receptor antagonism rescues stress-induced elevations in reticulocytes. Wildtype female C57Bl/6 mice were left unstressed (Control), subjected to 6 hrs of restrain stress (RST), subjected to RST and treated daily with 0.4 mg/day RU486 in 50 µL of 50% ethanol/50% PBS (RST + RU486), or subjected to RST and treated daily with 50 µL of 50% ethanol/50% PBS (RST + Vehicle). RU486 and vehicle treatments were given by subcutaneous injection at 0900h immediately before RST exposure. After 21 days, whole blood was collected by atrial puncture and circulating reticulocytes were quantified using an automated hematological cytometer. Reticulocytes from mice subjected to RST and RST mice treated with vehicle were significantly elevated compared to control mice and RST mice treated with RU486. Data were collected without repeated sampling of individuals. For each data point n = 5-8 individuals. Data shown is mean +SEM. *, p < .05 by ANOVA.

 RST drove increased numbers of circulating reticulocytes, which was rescued by the glucocorticoid receptor antagonist RU486 (Figure **5**).

### RST does not increase erythropoietin levels

**Figure 6:**
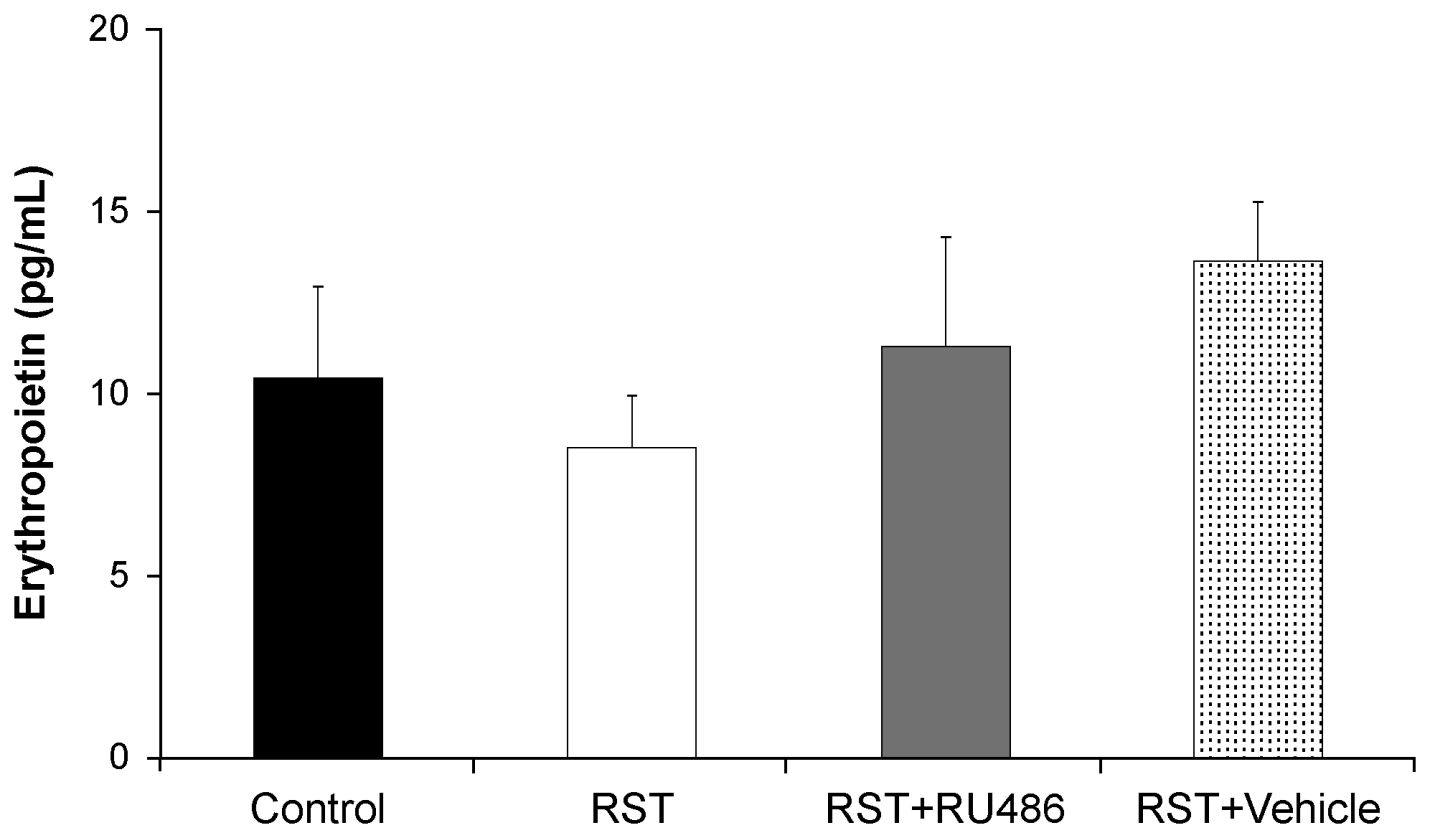
RST does not affect erythropoietin levels. Wildtype female C57Bl/6 mice were left unstressed (Control), subjected to 6 hrs of restrain stress (RST), subjected to RST and treated daily with 0.4 mg/day RU486 in 50 µL of 50% ethanol/50% PBS (RST + RU486), or subjected to RST and treated daily with 50 µL of 50% ethanol/50% PBS (RST + Vehicle). RU486 and vehicle treatments were given by subcutaneous injection at 0900h immediately before RST exposure. After 21 days, whole blood was collected by atrial puncture and serum levels of erythropoietin were quantified by ELISA. There was no statistical difference between mouse groups. For each data point, n = 5. Data shown is mean +SEM by ANOVA.

Because of the important role of erythropoietin in erythroid expansion, circulating erythropoietin levels were examined at Day 21. Erythropoietin levels remained within normal physiological ranges [33,34] under all conditions examined (Figure **6**).

### RST increases erythrocyte progenitor populations

**Figure 7:**
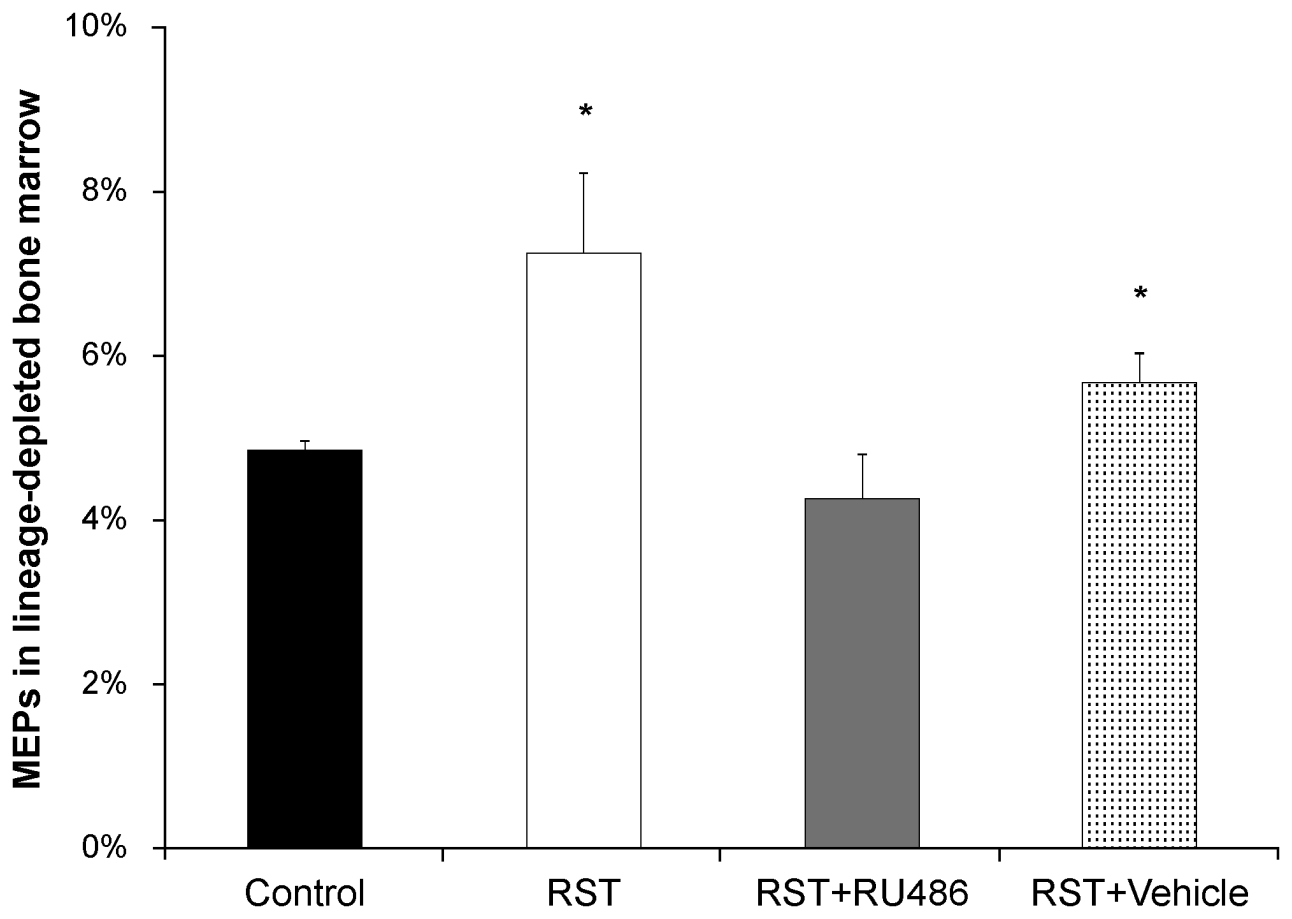
RST increases erythroid precursors in bone marrow, rescued by glucocorticoid receptor antagonism. Wildtype female C57Bl/6 mice were left unstressed (Control), subjected to 6 hrs of restrain stress (RST), subjected to RST and treated daily with 0.4 mg/day RU486 in 50 µL of 50% ethanol/50% PBS (RST + RU486), or subjected to RST and treated daily with 50 µL of 50% ethanol/50% PBS (RST + Vehicle). RU486 and vehicle treatments were given by subcutaneous injection at 0900h immediately before RST exposure. After 21 days and immediately following sacrifice, bone marrow was collected from femurs and immunostained with antibodies describing megakaryocyte-erythroid progenitor (MEP) cells (lin, CD34, CD16/32, Sca-1, and c-kit). The percent lin-/CD34-/CD16/32low/Sca-1-/c-kit+ MEP subpopulation relative to total cells were determined by flow cytometry. Mice subjected to RST had a significantly higher percentage of MEPs than unstressed control mice while RU486 suppressed elevation of MEPs in RST mice. For each data point, n = 5. Data shown is mean +SEM. *, p < 0.05 by ANOVA.

To determine the cause of increased erythroid expansion in response to RST, erythroid progenitor cells were quantified in bone marrow. Increased proportions of megakaryocyte-erythroid progenitor (MEP) cells were found at day 21 in mice exposed to RST and this effect was blocked by treatment with RU486 (Figure **7**).

### Progenitor differentiation favors erythrocyte formation in RST

**Figure 8:**
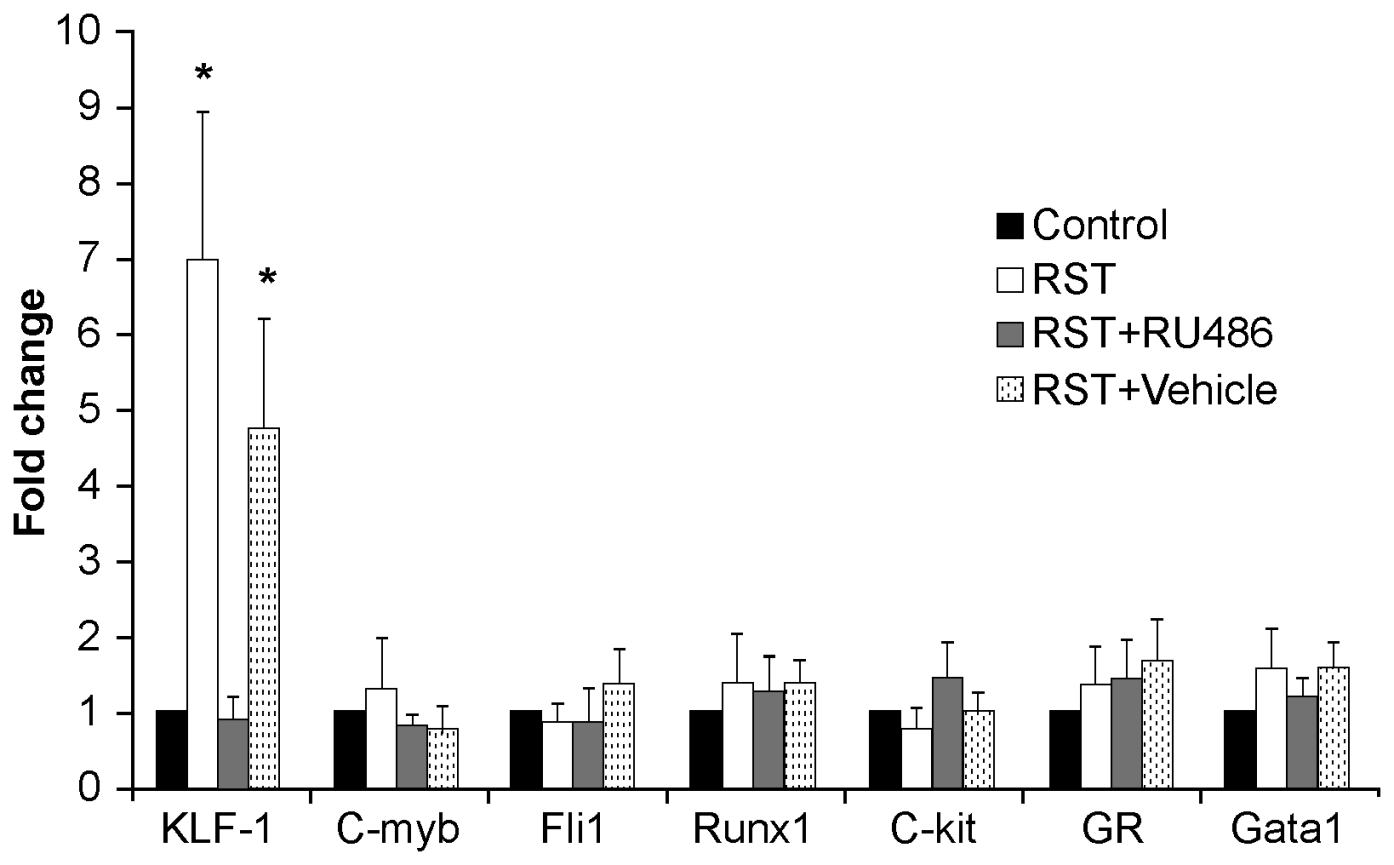
RST increases expression of *KLF1* in MEP cells, rescued by glucocorticoid receptor antagonism. Wildtype female C57Bl/6 mice were left unstressed (Control), subjected to 6 hrs of restrain stress (RST), subjected to RST and treated daily with 0.4 mg/day RU486 in 50 µL of 50% ethanol/50% PBS (RST + RU486), or subjected to RST and treated daily with 50 µL of 50% ethanol/50% PBS (RST + Vehicle). RU486 and vehicle treatments were given by subcutaneous injection at 0900h immediately before RST exposure. After 21 days and immediately following sacrifice, bone marrow was collected from femurs and immunostained with antibodies for lin, CD34, CD16/32, Sca-1, and c-kit. The lin-/CD34-/CD16/32low/Sca-1-/c-kit+ MEP subpopulation were collected by flow-assisted cell sorting. Cells were subjected to Trizol, RNA isolated and cDNA synthesized. Expression of the erythropoietic transcription factors (*KLF-1*, *c-myb*, *Fli1*, *Runx1*, *c-kit*, *Gata1*) and glucocorticoid receptor (GR) were examined by Real-time PCR. mRNA expression for all four mouse groups is expressed as fold change relative to the control group. Of all genes investigated, only *KLF-1* was significantly increased by RST. RU486 treatment abrogated RST-induced *KLF-1* levels similar to unstressed control mice. For each data point, n = 7-13. Data shown is mean +SEM. *, p < 0.05 by ANOVA for each mRNA in all four mouse groups.

To examine the role of glucocorticoids in regulating RST-driven erythropoiesis, expression levels of transcription factors involved in erythroid regulation of MEP cell fate were examined [17]. While many factors were evaluated, in agreement with microarray results only KLF1 was significantly elevated in response to RST (Figure **8**). Further, this increased KLF1 expression in response to RST was rescued by RU486 treatment. 

## Discussion

### Restraint stress elevates erythrocyte expression

Rodent restraint stress is regularly used in eliciting behavioral and biological symptoms associated with depressive disorders [35-38]. Clinical work reports finding reticulocytosis in individuals with major depression [39,40] and rodent studies describe increased reticulocyte counts in submissive mice exposed to social dominance [41]. Our laboratory reported on *in vivo* physiological and psychological responses to prolonged periods of chronic restraint stress [32]. Here mice exposed to an extended RST protocol demonstrate similar biological hallmarks of chronic restraint stress, namely, decreased body weight, spleen and thymus mass in conjunction with elevated circulating corticosterone and adrenal mass. These results align with findings in depressive stress models and distinguish from rodent models of acute stress [42,43] or anxiety [27,44]. To characterize the biological impact of extended psychological stress on genetic networks, genome-wide microarray analysis was performed on mice following seven days of RST. Importantly, work examining RNA expression profiles in breast cancer survivors suffering from chronic fatigue reveals elevated expression of genes linked to reticulocyte expression and hemoglobin synthesis [45]. Here, RNA expression analysis suggested disruption of hematological development in response to RST and biological pathway mapping predicted that many of these hematological changes are affected through the glucocorticoid receptor through the erythroid transcription factor KLF1.

With these findings in mind, mice were subjected to RST for up to 28 days and followed for up to 14 days following stress cessation. Coherent with microarray predictions, complete blood counts revealed increased numbers of circulating reticulocytes throughout the stress period which then returned to control levels following stress cessation. In considering the genesis of the observed increase in reticulocytes, we examined the formation of early terminally differentiated erythrocytes in primary and secondary hematopoietic tissues. During normal hematopoiesis long-bone marrow is the primary source of erythrocytes [28], however, in response to hypoxic, hemolytic, or chemical stress, extramedullary tissues such as spleen and liver serve as secondary sources of erythropoiesis [3,4]. In response to RST, mice experienced dramatic increases in the presence of the earliest terminally differentiated erythrocytes [29-31] in both primary and secondary hematopoietic tissues, demonstrating the ability of prolonged exposure to psychological stress to elicit increases in circulating reticulocytes as well as increased erythropoiesis in both primary and secondary hematopoietic organs.

### Restraint stress upregulates erythropoiesis through glucocorticoid elevations

Glucocorticoids play an integral role in the expansion and differentiation of erythroid progenitor cells. Despite this, little work has been undertaken to examine the effects of glucocorticoid elevations on erythropoiesis. While many studies have reported hematopoietic and even myeloid responses to psychological stressors [18-24], very little work has been done to describe erythroid changes in response to chronic psychological stress. Previous work in our laboratory has demonstrated that prolonged RST elicits elevated stress responses for up to 28 days [32] and chief among these is a sustained elevation in circulating corticosterone. To investigate the effects of corticosterone elevations on erythropoiesis, RST mice were treated with the glucocorticoid receptor (GR) antagonist RU486. All mice exposed to restraint stress demonstrated physiological and chemical markers of sustained stress responses following 21 days of RST. RU486-treated mice showed elevated stress responses similar to RST mice, indicating that GR antagonism did not diminish primary psychological or biological responses to RST-exposure. However, consistent with the hypothesis of glucocorticoid-driven elevations in erythropoiesis, GR antagonism provided a protective effect with regard to stress-induced elevations in circulating reticulocytes.

While Epo exerts tremendous influence on erythropoiesis, no elevations in circulating Epo levels were observed in response to RST or RU486 treatment and Epo levels for all conditions examined were found to be within the normal reported range [33,34]. Interestingly, while pharmacologic doses of cortisol have shown the inconsistent ability to elevate Epo in humans [46], psychological stress has been shown to be ineffective in influencing circulating Epo in rodents both here and elsewhere [47,48]. Instead, the ability of GR antagonism to rescue the stress-induced erythroid effects underscores the role of glucocorticoids in psychological stress-induced erythropoiesis.

### RST-induced erythropoiesis results from glucocorticoid-driven increases in erythroid progenitor populations

Glucocorticoids play an essential role in erythropoiesis both during fetal development and in homeostatic erythropoiesis [3,5-8] and insufficient cortisol production resulting from adrenal insufficiency associated with Addison’s disease causes anemia in humans [49]. Glucocorticoids enhance the formation of murine erythroid colonies [9] and increase erythroid proliferation under conditions of limiting Epo [10,11]. In fact, glucocorticoid stimulation induces long-term proliferation of erythroid precursors, capable of generating greater than 10-fold increases in pools of rapidly expanding erythroid progenitors [12,13]. Expression of KLF1 is restricted to erythroid cells and their precursors (megakaryocyte-erythroid progenitors, MEPs) and is essential in determining the erythroid- or megakaryocytic-fate of these bi-potent progenitors [16,17]. Activated GR acts with the transcription factor KLF1 to govern key transcriptional events that promote pro-erythroid transcription and ultimately guide MEP differentiation toward erythroid lineage [14-16,50]. Together, these findings suggest that sustained elevations in glucocorticoid levels evoked by extended periods of psychological stress might exert a positive influence on erythropoiesis. Accordingly, this work showed that (i) RST increased populations of erythroid precursors in the bone marrow and (ii) this effect was blocked by treatment with the GR antagonist, indicating that (iii) increased numbers of erythroid progenitors were produced in response to elevations in glucocorticoid levels evoked by prolonged RST. Previous work has demonstrated the direct effect of glucocorticoids on expansion of erythroid progenitors *in vitro* [12,13] though *in vivo* experiments have yielded varying results [51,52]. To our knowledge this is the first evidence of elevation in the erythroid progenitor populations in response to psychological stress.

Analysis of key transcriptions factors directing MEP development and differentiation corroborated microarray results, indicating increased expression of the pro-erythroid transcription factor KLF1 in response to RST, an effect which was blocked by GR-antagonism. While decisions affecting self-renewal and differentiation are guided by many key cellular interactions, the role of KLF1 in determining the fate of MEPs is essential and definitive [53-55]. The ability to block these responses through treatment with a GR antagonist indicates the observed effects are produced in response to RST-induced increases in glucocorticoid levels. Glucocorticoid-dependent upregulation of KLF1, increased MEP populations, increased terminal erythroid formation in hematopoietic organs, and increases in circulating reticulocytes observed here are coherent with initial predictions based on microarray analysis. In addition to introducing the topic for future studies on additional downstream erythropoietic responses, these findings broaden the application of predictive genomics and increase our understanding of the physiological consequences of chronic psychological stress.

## Supporting Information

Figure S1 **Flow cytometry parameters used for identification of CD71^+^/Ter119^+^ erythrocytes.**
(TIF)

Figure S2 **Primers used for RT-PCR.**
(TIF)
