# Supplementary figures and images for: Chronic Restraint Stress Upregulates Erythropoiesis through Glucocorticoid Stimulation
Source: PLoS One. 2013 Oct 18;8(10):e77935. doi: 10.1371/journal.pone.0077935 (PMC3799740; doi:10.1371/journal.pone.0077935)

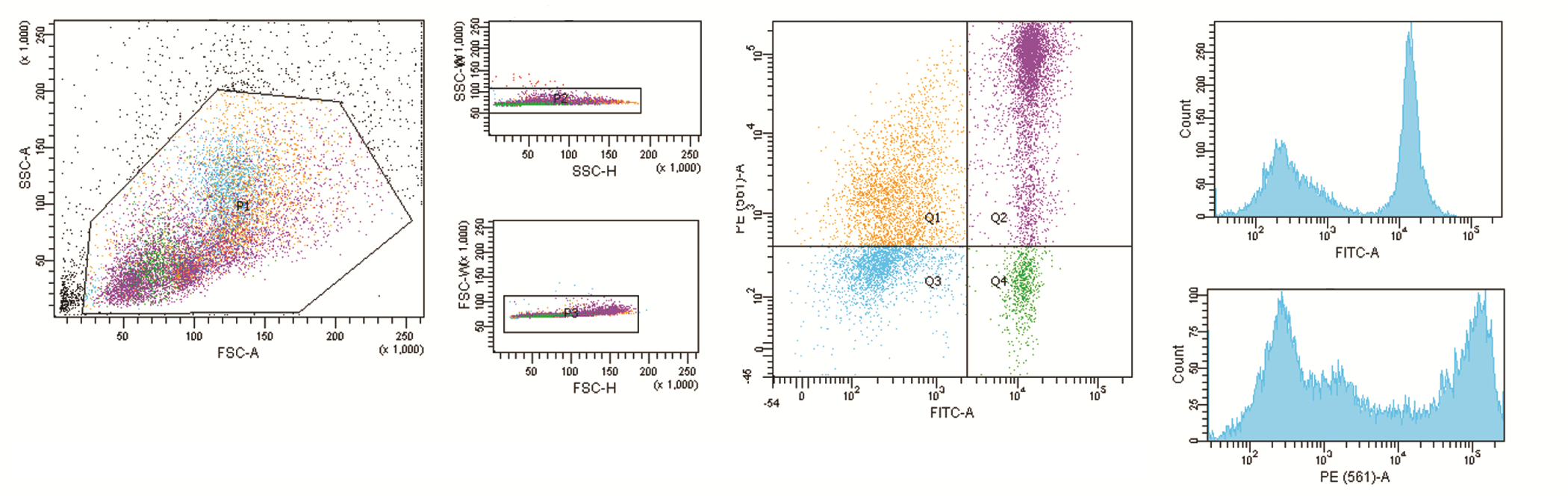

Supplement: Figure S1 — Flow cytometry parameters used for identification of CD71+/Ter119+ erythrocytes. (TIF) [file pone.0077935.s001.tif]
